# Gp41 and *Gag* amino acids linked to HIV‐1 protease inhibitor‐based second‐line failure in HIV‐1 subtype A from Western Kenya

**DOI:** 10.1002/jia2.25024

**Published:** 2017-11-03

**Authors:** Mia Coetzer, Lauren Ledingham, Lameck Diero, Emmanuel Kemboi, Millicent Orido, Rami Kantor

**Affiliations:** ^1^ Division of Infectious Diseases Brown University Providence RI USA; ^2^ Academic Model Providing Access to Healthcare (AMPATH) Eldoret Kenya; ^3^ Department of Medicine School of Medicine College of Health Sciences Moi University Eldoret Kenya

**Keywords:** HIV‐1, protease, drug resistance, gag, gp41, subtype A, Kenya

## Abstract

**Introduction:**

Failure of protease‐inhibitor (PI)‐based second‐line antiretroviral therapy (ART) with medication adherence but no protease drug resistance mutations (DRMs) is not well understood. This study investigated the involvement of gp41 and *gag* as alternative mechanisms, not captured by conventional resistance testing and particularly relevant in resource‐limited settings where third‐line ART is limited.

**Methods:**

We evaluated gp41 and g*ag* for unique amino acids in seven subtype A infected Kenyans failing second‐line therapy with *no* PI resistance yet detectable lopinavir (query dataset), compared to seven similar‐setting patients *with* PI resistance or undetectable lopinavir and 69 publically available subtype A Kenyan whole‐genomes sequences.

**Results:**

Three gp41 (607T, 641L, 721I) and four *gag* (124S, 143V, 339P, 357S) amino acids were significantly more frequent in the query dataset compared to the other datasets, with significantly high co‐occurrence.

**Conclusion:**

The genotypic analysis of a unique group of HIV‐1 subtype A infected patients, identified seven amino acids that could potentially contribute to a multi‐gene mechanism of PI‐based ART failure in the absence of PI DR mutations.

## 1 Introduction

The main cause of HIV‐1 antiretroviral therapy (ART) failure is drug resistance (DR) development. In resource‐limited settings (RLS) there are only two recommended ART lines: a non‐nucleoside reverse transcriptase inhibitor (NNRTI) based first‐line, and a boosted protease inhibitor (PI) based second‐line, each with two nucleoside reverse transcriptase inhibitors (NRTIs). However, already observed second‐line failure is estimated to escalate with limited third‐line options 1. Detection and understanding second‐line DR in RLS is therefore important to sustain treatment efficacy and plan for effective future therapy.

Failure of second‐line ART in RLS, typically based on lopinavir/ritonavir (LPV/r), is seen at an average of 30% 1. Known PI DR mechanisms involve the development of mutations in and around the protease active site that change its interaction with the inhibitor 2. Such changes typically lead to fitness costs and loss of replication capacity that can be compensated by more distant protease mutations 3. However, whereas >70% of patients who fail first/second‐line failure have reverse transcriptase (RT) DR mutations (DRMs), patients failing second‐line ART have low levels (average 18%) of PI DR 4, which is not well understood. This observation suggests alternative DR mechanisms that lead to PI DR, such as (1) non‐adherence or decreased PI levels 5, 6, (2) resistant minor variants not detected by conventional assays 7, or (3) regions outside the protease such as *gag* 8, with increasing data suggesting its importance, and the recently suggested gp41 in *envelope (env)* 9.

To further understand low PI DR upon second‐line ART failure and potentially associated alternative genomic regions, we focused on the gp41 region and investigated whether a unique cohort of HIV‐1 infected Kenyans failing second‐line ART with no protease DRMs, but detectable LPV levels, have amino acids that could potentially be associated with PI DR, while also examining the *gag* gene.

## 2 Methods

Plasma samples from Kenyan adults participating in a study at the Academic Model Providing Access to Healthcare (AMPATH) in Eldoret, Kenya 10 were selected if they were (1) infected with HIV‐1 subtype A (by *pol*), the most common subtype in Kenya, (2) failing LPV/r‐based second‐line ART after >6 months, with prior >6 months NNRTI‐based first‐line ART, (3) had detectable plasma LPV and (4) had no protease DRMs. This “Query Dataset,” hypothesized to have alternative PI DR mechanisms, was compared to a “Background Dataset,” obtained from AMPATH patients from the same study, eligible for criteria (1) to (3) above, but *with* PI DRMs or with *undetectable* LPV levels, in which alternative PI DR mechanisms were less likely. At the time of this study monitoring of patients on ART was mostly immunological‐ or clinical‐based. VL or drug resistance testing was limited and therefore the precise cause of treatment failure was unknown.

CD4 (FACSCalibur platform; BD‐Biosciences San Jose, CA) and viral load testing (Amplicor; Roche Molecular, Pleasanton, CA) were performed at the AMPATH Laboratory; *pol* genotyping at the Providence‐Boston Center for AIDS Research; and LPV levels (liquid chromatography/tandem mass spectrometry) at the University of North Carolina Center for AIDS Research.

Viral RNA was extracted (EZ1 Advanced system; Qiagen, Hilden, Germany), followed by cDNA generation using reverse primer nef‐O‐R (5′‐aggcaagctttattgagg‐3′, HXB2 nucleotides [nt] 9625‐9608) and SuperscriptIII First Strand Synthesis System (Thermofisher, Carlsbad, CA, USA) per manufacturer's instructions. cDNA was subjected to first‐round PCR using Phusion High‐Fidelity DNA Polymerase (New England Biolab, Ipswich, MA, USA) with gp41‐primers envA (5′‐ggcttaggcatctcctatagcaggaagaa‐3′, nt 5950‐5982) and envM (5′‐tagcccttccagtccccccttttctttta‐3′, nt 9068 to 9096); and *gag*‐primers msf12 (5′‐aaatctctagcagtggcgcccgaacag‐3′, nt 623 to 649) and 2610R (5′‐ttcttctgtcaatggccattgtttaac‐3′, nt 2610 to 2636); followed by a second PCR using gp41‐primers V3‐inner (5′‐cagtacaatgtacacatggaatt‐3′, nt 6955 to 6977) and envM; and *gag* primers WGPf1 (5′‐tctctcgacgcaggactcg‐3′, nt 682 to 700) and DSPR (5′‐gggccatccattcctggc‐3′, nt 2588 to 2605). Sanger sequencing was performed on positive PCR products and submitted to Genbank (accession numbers KY351787‐KY351810). Ethics approvals were obtained from Lifespan and AMPATH Committees.

To compensate for the small sample sizes of the Query and Background Datasets and to further evaluate the presence of unique gp41 and/or *gag* amino acids associated with PI DR, two additional Kenyan, HIV‐1 subtype A, full‐genome datasets were compiled from the Los Alamos Database (http://www.hiv.lanl.gov): (1) an “ART‐Naïve Dataset”, sequences from literature‐confirmed ART‐naïve patients; and (2) a “Population Dataset”, all remaining sequences, from patients with unclear ART histories but mostly from earlier (<2002) studies before PI introduction to Kenya. Insignificant presence of unique gp41 and/or *gag* amino acids in these datasets as compared to the Query Dataset would support their potential involvement in alternative PI DR mechanisms, rather than being subtype‐specific polymorphisms or evolutionary‐associated changes.

Within the four datasets (Query, Background, Naïve and Population), *pol* DRMs were detected according to the IAS‐USA 2015 mutation list with Stanford HIV Database tools (hivdb.stanford.edu). The gp41 and *gag* sequences were aligned with BioEdit v.7 (http://www.mbio.ncsu.edu/BioEdit). Phylogenetic analysis was performed by maximum‐likelihood using Mega5 (http://www.megasoftware.net), including sequences from the HIV‐1 Subtype Reference Alignment (Los Alamos Database), confirming subtype designation and lack of epidemiological linkage.

We hypothesized that Query Dataset viruses have unique amino acids outside *pol* that may be associated with PI failure, and that these are not common in patients from the same cohort with PI DRMs or lacking adherence as represented by undetectable LPV (Background Dataset), or in the Naïve or Population Datasets. To test this hypothesis we first identified gp41 and/or *gag* signature amino acids that differ between the Query and the Background Datasets, which are from the same Kenyan setting. The Los Alamos Viral Epidemiology Signature Pattern Analysis (VESPA) tool was used to identify signature amino acids, defined as positions in which most common amino acids differ among two datasets. A threshold of zero was used, selecting the common amino acid regardless of its frequency. Second, we identified which of these signature amino acids also differ between the Query and the Naïve, and the Query and Population Datasets. Third, we compared proportions of these signature amino acids between the Query Dataset and the three other datasets using Fisher exact tests (*p*<0.05 considered significant). Lastly, we did similar comparisons between the Background Dataset and the Naïve and Population Datasets, to explore the presence of these amino acids within the Kenyan population.

## 3 Results

Samples from 14 eligible patients were available (median CD4 count 115 cells/μl and viral load 48,800 copies/ml), seven each in the Query and Background Datasets (Table 1). All Query Dataset patients had NRTI and/or NNRTI, but no PI DRMs despite detectable LPV. In the Background Dataset three patients had major PI DRMs, two with detectable and one with undetectable LPV; and four had no PI DRMs with undetectable LVP. For comparison, DRMs were minimal in the Naïve Dataset (NNRTI‐associated V179T (n=2) and E138A (n=1)); and the Population Dataset (PI‐associated M46L (n=1), NRTI‐associated K65R (n=1), NNRTI‐associated V106I (n=1) and E138A (n=2)).

**Table 1:** Demographic, laboratory, *Pol*‐resistance and lopinavir‐level characteristics of HIV‐1 subtype A infected AMPATH patients failing second‐line PI‐based ART in the Query (ID PI001 to PI007) and Background (ID PI008 to PI014) Datasets

ID	Age	Sex	Second‐line regimen	Time on second‐line regimen (years)	CD4 count (cells/μl)	VL (copies /ml)	PI	NRTI	NNRTI	LPV level (ug/mL)
PI001	53	M	ABC,DDI,	2.9	100	181,000	None	None	K103KN	1.17
			LPV,RTV							
PI002	41	M	ABC,DDI,	0.7	155	153,700	None	None	K103KNRS, G190A	>10
			LPV,RTV							
PI003	18	M	ABC,DDI,	0.6	10	64,500	None	K65R, K219EK	K103N, Y181CY	>10
			LPV,RTV							
PI004	31	F	3TC,TDF,	0.9	40	15,900	None	K65R, T69d, K219R	K103EK, Y181V	2.24
			LPV,RTV							
PI005	21	F	3TC,TDF,	0.5	10	14,600	None	T69NS	G190A	3.55
			LPV,RTV							
PI006	63	M	AZT,DDI,	1.2	175	9500	None	D67DG, K70R, K219Q	Y181C	0.77
			LPV,RTV							
PI007	55	M	ABC,DDI,	2.5	300	4500	None	M41L, D67N, V75I, M184V, L210W, T215F	K103N	>10
			LPV,RTV							
PI008	50	M	AZT,DDI,	7.3	305	32,000	M46I, I54V, V82A, L90M	M41L, D67N, L74V, M184V, L210W, T215Y	G190A	7.2
			LPV,RTV							
PI009	34	F	AZT,DDI,	4.6	10	55,200	V32I, M46LM, I47A, L90LM	M41LM, D67N, T69NT, K70R, T215FIST, K219Q	None	6.57
			LPV,RTV							
PI010	31	F	ABC,DDI,	0.9	165	42,400	M46I, I54IV, V82A	M184V, T215Y	V108IV, Y181C	BLD
			LPV,RTV							
PI011	38	M	AZT,DDI,	3.5	340	5100	None	K70KR, M184MV	K103N, P225HP, K238KT	BLD
			LPV,RTV							
PI012	43	F	ABC,DDI,	0.9	90	74,300	None	None	None	BLD
			LPV,RTV							
PI013	38	F	3TC,TDF,	0.5	130	820,700	None	None	None	BLD
			LPV,RTV							
PI014	44	M	ABC,DDI,	0.5	35	95,000	None	None	G190AG	BLD
			LPV,RTV							

3TC, lamivudine; ABC, abacavir; AZT, zidovudine; DDI, didanosine; RTV, ritonavir; TDF, tenofovir; T69d, deletion; BLD, below the level of detection; CD4 count rounded to the closest 5; VL rounded to closest 100.

Generated sequences included 6/7 gp41 and 7/7 *gag* in the Query and 5/7 gp41 and 6/7 *gag* in the Background Datasets. Phylogenetic analysis of all gp41 and *gag* sequences confirmed HIV‐1 subtype A designation, with no epidemiologic linkage (data not shown).

VESPA identified 24 gp41 positions where amino acids differed between the Query and Background Datasets. Of those 24, 14 also differed between the Query and Naïve Datasets. At three of the 14 positions the amino acids were significantly higher in the Query compared to the Background (607T) or the Naïve Dataset (607T, 641L, 721I). These three amino acids were also signature in the Query compared to the Population Dataset, two significantly higher (607T, 721I) (Table 2).

**Table 2:** Proportions of unique gp41 and *gag* amino acids in the four datasets and their co‐occurrence in the Query Dataset

	*gag*				gp41		
	124S	143V	339P	357S	607T	641L	721I
Unique amino acid proportions in the four datasets							
Query	0.71	0.43	0.57	0.57	0.83	0.5	0.5
Background	0.29	0.29	0.29	0.43	<0.01*	0.4	0.2
Naïve	0.30*	0.13**	0.13*	0.13*	0.13*	0.03*	0.06*
Population	0.3	0.19	0.3	0.38	0.11*	0.24	0.03*
Unique amino acid co‐occurrence in the Query Dataset							
Sample ID							
PI001	S			S		L	
PI002					T	L	I
PI003	S	V	P	S	T	L	
PI004	S	V	P	S	T		I
PI005					T		
PI006	S		P	S	na		
7	S	V	P		T		I

*p<0.05 and **p=0.06 for comparisons with the Query Dataset. na, not available.

Similar *gag* analyses resulted in 17 positions identified by VESPA where common amino acids differed between the Query and Background Datasets, 11/17 also different between the Query and Naive Datasets. At four of these 11 sites amino acids were significantly higher in the Query compared to the Naïve Dataset (124S, 143V, 339P and 357S), and although they were not significantly more prevalent compared to the Background and Population Datasets they were selected for further analysis (Table 2). These four amino acids were also signature in the Query compared to the Population Dataset. *Gag* was also examined for previously described HIV‐1 subtype B‐specific cleavage site (CS: MA/CA 128I/T/A, NC/p1 431V, 436E/R and 437T/V; and in p1/p6‐*gag* 449F/P/V, 452S/K and 453A/L/T) and non‐CS (R76K, Y79F and T81A) mutations, associated with either PI DR or exposure 8. Although three CS mutations (128del, 436R and 449P) and two non‐CS mutations (76K and 79F) were present in the Query Dataset they were similarly common (>30%) and not significantly different from any other dataset, and 449P was conserved in all sequences from the four datasets suggesting a subtype A‐associated *gag* polymorphism. The occurrence of previously described *gag* CS and non‐CS mutations in the Naïve Dataset, further highlight the possible role of subtype‐specific *gag* variability and the potential for multiple pathways to PI failure.

In similar comparisons of the Background and Naïve, or the Background and Population Datasets, four of the four *gag* mutations and two of the three gp41 mutations did not differ between the datasets, supporting our hypothesis. One mutation, gp41 641L, occurred more in the Background (2/5) than in the Naïve Dataset (1/31; *p*=0.04). One of these two Background Dataset patients had a detectable LPV level with three‐class DR, still potentially supporting the alternative DR mechanism hypothesis. The other patient had an undetectable LPV level and no PI DR, suggesting the need for larger numbers and further characterization.

Co‐occurrence of any of the identified three gp41 and four *gag* amino acids in both genes was detected in 4/6 (67%) patients in the Query Dataset (detailed in Table 2), 1/5 (20%, *p*=0.24) in the Background Dataset, 2/32 (6%, *p*<0.01) in the Naive Dataset and 11/37 (30%; *p*<0.01) in the Population Dataset. Six of the 7 (86%) patients in the Query Dataset had ≥3 of these amino acids (in any gene), compared to the Background 2/7 (29%, *p*=0.10), Naïve 2/32 (6%, *p*<0.01) and Population 5/37 (14%, *p*<0.001) datasets. Examination of co‐occurrence of the gp41 and/or *gag* mutations with major PI DRMs was limited to four samples: three from the Background Dataset, one (PI008) with a gp41 (641I) and *gag* (143V) mutation, and two (PI009 and PI010) with one *gag* mutation each (124S and 357S respectively); and one from the Population Dataset with a *gag* (357S) mutation, suggesting that the gp41 and *gag* mutations could be interconnected with well‐defined PI DRM.

## 4 Discussion

This study investigated alternative DR mechanisms in a small but unique Kenyan cohort of HIV‐1 subtype A infected adults failing PI‐based second‐line ART with a particular focus on gp41. We identified three novel amino acids that were significantly more prevalent in individuals with no protease DRMs but detectable LPV levels, as compared to ART naïve Kenyans: two in the heptad repeat region (607T, 641L) and one in the cytoplasmic tail (721I) of gp41. We also found a higher prevalence of four *gag* mutations, one in the matrix (124S) and three in the capsid (143V, 339P, 357S) structural proteins of *gag*. Though these findings may suggest a potential role for these amino acids in new mechanisms of PI resistance, any hypothesis on their actual involvement in alternative mechanisms is speculative.

Current research suggests that PIs do not only block the viral protease activity, but may also affect viral entry that is facilitated by *env* 9. In that study gp41 conferred PI resistance, whereas *gag* and *pol* had no PI‐associated DRMs. The authors' proposed an alternative resistance mechanism that includes amino acids within *env* that can overcome PI‐mediated inhibition of viral entry, by changing the interaction between the cytoplasmic tail of gp41 and the uncleaved *gag*. One candidate gp41 amino acid identified here is in the cytoplasmic tail further supporting this suggested alternative mechanism. The other two amino acids are in the heptad repeat regions, important in viral fusion. However, as also noted by Rabi *et al*., identification of amino acids within gp41 that might confer PI resistance is complicated by the high diversity within this region, requiring more genotypic and phenotypic analysis.

Studies regarding the involvement of regions outside the protease in PI DR have shown that *gag* CS and non‐CS mutations might be associated with PI failure with or without PI DRMs, restoring viral replication capacity, increasing viral fitness and improved protease binding affinities for the mutant *gag* substrate 8, 11. Substitutions in *gag* have also been linked to reduced PI drug sensitivity in the presence of wild‐type protease 12, 13, highlighting the need to possibly include *gag* in drug susceptibility assays. A recent study showed that mutations in *gag* can develop and accumulate overtime and contribute independently to drug resistance, or lead to further mutation development within *protease* 14. Similar investigations including, for example, mutagenetic tree analysis to determine the interconnection between *gag* and these mutations, would be important, but not performed here due to limited numbers. Notably, most studies addressing genetic variation within *gag* have focused on HIV‐1 subtype B samples, with available data suggesting more variation within non‐B subtypes 15. The new *gag* amino acids identified here could contribute in a similar way, though this needs to be determined. Further investigation such as phenotypic confirmation of the amino acid contribution is urgently needed to validate our findings and determine the significance, mechanism and impact of these candidate gp41 and *gag* amino acids in PI‐based second‐line ART failure. Subtype A was examined here as it is the most common HIV‐1 variant in Kenya (approximately 70%) 16. However, the presence and role of these amino acids in other subtypes as well as their relation to other PIs warrant further investigation.

Caveats of this study include, first, the need for phenotypic confirmation of the candidate amino acids' role during PI treatment failure, either as independent or compensatory mutations; second, the availability of only a single LPV level measurement, not adequately reflecting adherence 17; third, the use of population sequencing without consideration of minor resistance variants, which may interfere with ART effectiveness 7; and lastly, though patients were unique, samples sizes were small, limiting power and generalizability and necessitating widening of the datasets to Kenyan but non‐AMPATH settings, with at times less complete treatment histories and more limited interpretation.

## 5 Conclusion

Genotypic analyses of a small yet unique cohort of subtype A infected patients from western Kenya identified seven amino acids in gp41 and *gag* that could potentially contribute to a multi‐gene mechanism of PI‐based ART failure in the absence of PI DR mutations. The alternative PI DR pathways investigated here, which require larger numbers and phenotypic validation, will most probably not be exclusively responsible for failure of PI‐based second‐line ART in the absence of PI DR. However, since HIV‐1 gp41 and *gag* are not routinely analysed phenotypically or genotypically, identification of alternative mechanisms involving these genes is limited. Data presented here propose potential avenues for further investigation of such mechanisms. Such information is required to improve treatment monitoring and DR interpretation, particularly in RLS, as well as to conduct strategic planning for third‐line ART options.

## Competing interest

All authors declare no competing interest for this work.

## Authors' contributions

MC and RK conceived and developed the study and wrote the manuscript. RK and LD designed and supervised the study protocol. EK and LD enrolled patients. MC, LL and MO conducted laboratory assays. MC and LL analysed data. All authors have read and approved the final manuscript.

## Funding Sources

This work was supported by National Institute of Health grants P30AI042853 (Providence‐Boston Center for AIDS Research) and AI108441; and the Rhode Island Foundation (20123826).
